# Inhibiting DNA-PKcs in a non-homologous end-joining pathway in response to DNA double-strand breaks

**DOI:** 10.18632/oncotarget.15153

**Published:** 2017-02-07

**Authors:** Jun Dong, Tian Zhang, Yufeng Ren, Zhenyu Wang, Clifton C. Ling, Fuqiu He, Gloria C. Li, Chengtao Wang, Bixiu Wen

**Affiliations:** ^1^ Department of Radiation Oncology, The First Affiliated Hospital, Sun Yat-sen University, Guangzhou 510080, China; ^2^ Department of Medical Physics and Radiation Oncology, Memorial Sloan-Kettering Cancer Center, New York, NY 10021, USA

**Keywords:** DNA-PKcs, non-homologous end-joining, double-strand break, NU7441, nasopharyngeal carcinoma

## Abstract

DNA-dependent protein kinase catalytic subunit (DNA-PKcs) is a distinct factor in the non-homologous end-joining (NHEJ) pathway involved in DNA double-strand break (DSB) repair. We examined the crosstalk between key proteins in the DSB NHEJ repair pathway and cell cycle regulation and found that mouse embryonic fibroblast (MEF) cells deficient in DNA-PKcs or Ku70 were more vulnerable to ionizing radiation (IR) compared with wild-type cells and that DSB repair was delayed. γH2AX was associated with phospho-Ataxia-telangiectasia mutated kinase (Ser1987) and phospho-checkpoint effector kinase 1 (Ser345) foci for the arrest of cell cycle through the G2/M phase. Inhibition of DNA-PKcs prolonged IR-induced G2/M phase arrest because of sequential activation of cell cycle checkpoints. DSBs were introduced, and cell cycle checkpoints were recruited after exposure to IR in nasopharyngeal carcinoma SUNE-1 cells. NU7441 radiosensitized MEF cells and SUNE-1 cells by interfering with DSB repair. Together, these results reveal a mechanism in which coupling of DSB repair with the cell cycle radiosensitizes NHEJ repair-deficient cells, justifying further development of DNA-PK inhibitors in cancer therapy.

## INTRODUCTION

Cell cycle checkpoints are governed primarily by the kinase Ataxia-telangiectasia mutated (ATM) and Ataxia-telangiectasia and Rad3-related (ATR). DSB results in activation of ATM, which triggers phosphorylation of checkpoint effector kinase (CHK2), leading to the arrest of G1/S checkpoints, whereas the intra-S phase or G2/M checkpoints are blocked by CHK1 and activated by ATR. There is strong crosstalk between the ATM–CHK2 and ATR–CHK1 pathways, which relays and thus amplifies the cellular damage signal by phosphorylating many DNA damage response (DDR) proteins [4, 8–11].

DNA-dependent protein kinase catalytic subunit (DNA-PKcs), a member of the phosphatidylinositol 3-kinase (PI3K) family, represents a central component in NHEJ repair. DNA-PKcs is recruited to the DSB site and binds to the Ku70/80 heterodimer to form the DNA repair complex, which activates the kinase activity of DNA-PKcs through phosphorylation of the serine/threonine [12]. Our previous studies have validated that the absence of Ku protein compromises the ability of cells to repair DSB via the NHEJ repair pathway, elevates radiosensitivity, and enhances radiation-induced apoptosis [13, 14].

Studies have suggested a connection between reduced levels of ATM and alleviated levels of DNA-PKcs, which may rely on downstream substrates after exposure to IR [15, 16]. G2/M phase arrest arises and persists even at 24 to 48 h post-irradiation by inhibition of DNA-PKcs in ATM-deficient cells [15]. These data indicate the involvement of DNA-PKcs in DSB repair and its regulatory role in cell cycle progression.

There are comprehensive links among DNA repair, cell cycle progression and genome integrity in different cell lines. In radioresistant prostate cancer cells, the combination of PI3K/mTOR inhibitor with irradiation suppresses colony formation, enforces G2/M phase arrest and maintains high levels of DSB [17]. Cisplatin radiosensitizes non-small cell lung cancer cells via phosphorylation of ATM and autophagy [18]. However, the mechanism of DSB repair involved in NHEJ mediated by cell checkpoint(s) still remains unclear.

Here, using NHEJ-deficient cells in which DNA-PKcs and Ku70 genes were knocked down, we sought to gain mechanistic insights into the crosstalk between key proteins involved in the DSB NHEJ repair pathway and cell cycle regulation. We used a DNA-PK inhibitor to explore the synergistic effect on inhibition of DSB repair following IR exposure in NHEJ-competent or -deficient cells and a cancer cell line from a nasopharyngeal carcinoma (NPC) patient.

## RESULTS

### Deficiency of DNA-PKcs increases sensitivity to irradiation

**Figure 1:**
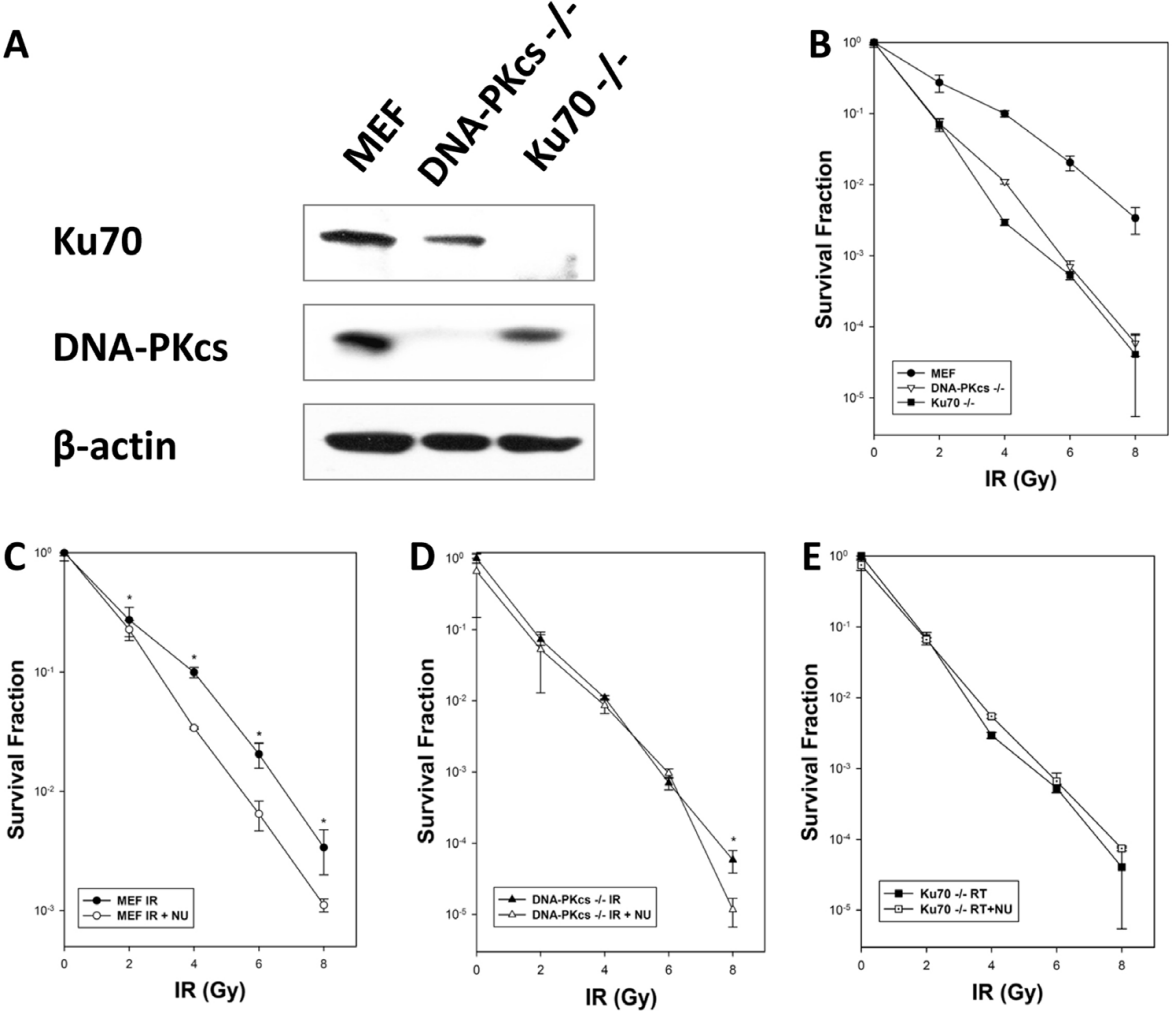
Cellular radiosensitivity depends on NHEJ after exposure to ionizing radiation (IR) (**A**) Western blot analysis showed expression of Ku70 and DNA-PKcs proteins in MEF, DNA-PKcs^−/−^ MEF, and Ku70^−/−^ MEF cells. (**B**) MEF, DNA-PKcs^−/−^ MEF, and Ku70^−/−^ MEF cells were treated with various X-ray doses. MEF (**C**), DNA-PKcs^−/−^ (**D**), and Ku70^−/−^ (**E**) cells were exposed to IR alone or in combination with NU7441 (1 μM) for 16 h before seeding for colony formation. Clonogenic survival data are means of three independent experiments ± SD.

To investigate the role of DNA-PKcs in response to IR, we used mouse embryonic fibroblast (MEF), DNA-PKcs^−/−^ MEF, and Ku70^−/−^ MEF cell lines to evaluate their differences in radiosensitivity. Western blot analysis was conducted to reveal the loss of Ku70 protein in Ku70^−/−^ cells and DNA-PKcs protein expression in DNA-PKcs^−/−^ cells (Figure 1A). All cell lines were exposed to irradiation at graded doses (0, 2, 4, 6, and 8 Gy), and survival was determined using the colony formation assay. The results indicated that irradiation induced a reduction of cell survival probability in DNA-PKcs^−/−^ and Ku70^−/−^ cells compared with their wild-type MEF cells (Figure 1B).

NU7441, a specific DNA-PK inhibitor, was used to investigate any synergistic effect when combined with irradiation in MEF, DNA-PKcs^−/−^ MEF and Ku70^−/−^ MEF cell lines. Our data showed that the combination of IR and NU7441 (1 μmol/L) significantly stimulated cell death in MEF cells when compared with IR alone but had a modest effect on DNA-PKcs^−/−^ and Ku70^−/−^ cells (Figure 1C–1E), supporting that potentiation was attributable to DNA-PKcs or Ku70 inhibition. Ku70 is an acknowledged NHEJ repair pathway protein and deletion of Ku70 significantly decreases cell viability by hindering DSB repair [13]. Therefore, we speculated that cells with knockdown of DNA-PKcs were susceptible to IR because of unrepaired DSBs.

### Ionizing radiation induces γH2AX formation and loss of DNA-PKcs, or Ku70 delays DSB repair

**Figure 2:**
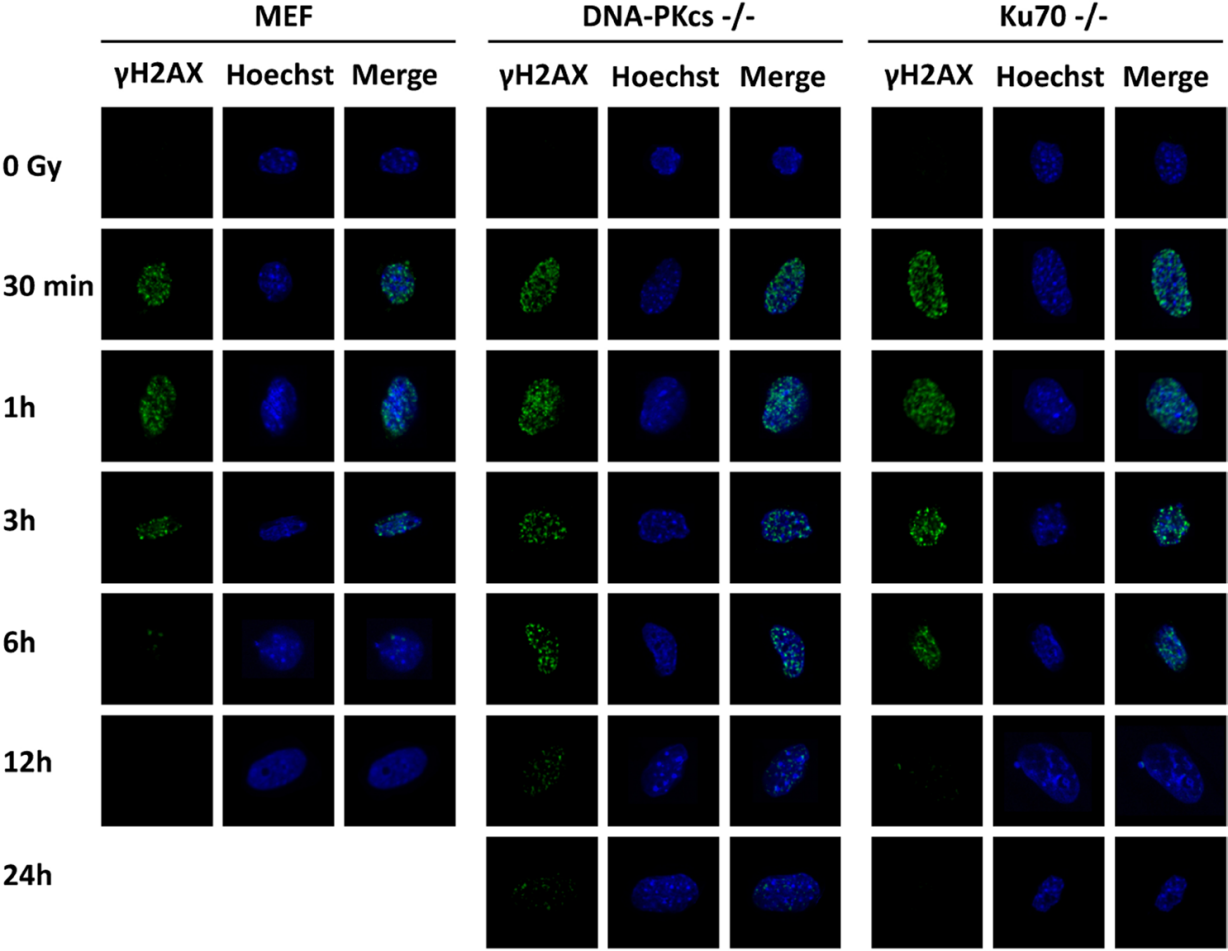
γH2AX foci formation is detected in MEF, DNA-PKcs^−/−^ MEF, and Ku70^−/−^ MEF cells γH2AX foci were formed immediately after 5 Gy X-ray and the resolution of it delayed in NHEJ repair-deficient cells. Representative photomicrographs (×1000 magnification) shown.

DNA-PKcs participates in multiple aspects of the NHEJ process in DSB repair [12]. γH2AX serves as a useful surrogate for unrepaired DSB, and its dispersal correlates with DSB repair [19, 20]. We investigated the capacity of DSB repair in MEF, DNA-PKcs^−/−^ MEF, and Ku70^−/−^ MEF cells after exposure to irradiation using γH2AX foci formation analysis at specific time intervals. Our data showed that γH2AX foci formation was evident and abundant at 30 min, peaked at 1 h and then fell gradually in MEF cells (Figure 2). It was similar to MEF cells at 0.5 and 1 h post-IR for DNA-PKcs^−/−^ and Ku70^−/−^ MEF cells, whereas the number of γH2AX foci per cell persisted at a higher level at 3 h post-IR in DNA-PKcs^−/−^ and Ku70^−/−^ MEF cell lines. Moreover, the kinetics of disappearance of γH2AX foci was much slower in DNA-PKcs^−/−^ and Ku70^−/−^ MEF cells than in MEF wild-type cells, implying that an inappropriate NHEJ pathway compromised the ability of cells to repair DNA DSBs (Supplementary Figure 1).

### Magnitude of unrepaired DSBs is associated with phosphorylation of ATM and CHK1

**Figure 3:**
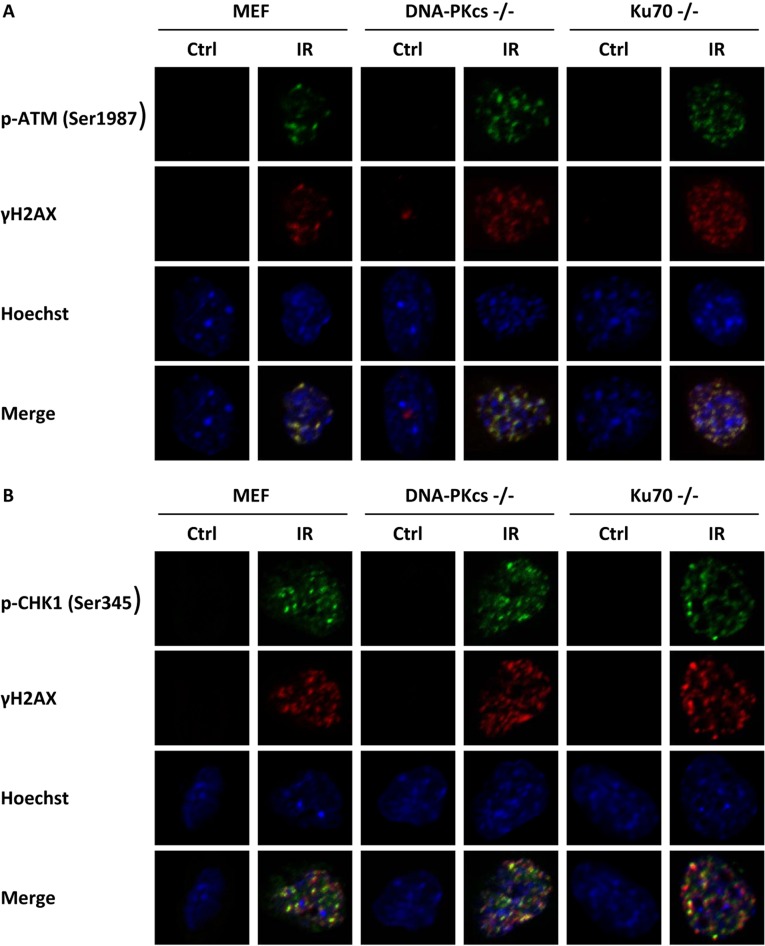
γH2AX is associated with phosphorylation of ATM and CHK1 MEF cells with knockdown of DNA-PKcs and Ku70 were irradiated with 5 Gy X-ray and co-immunofluorescence stained with γH2AX antibody and p-ATM (Ser1987) antibody (**A**), or with γH2AX antibody and p-CHK1 (Ser345) antibody (**B**) at 1 h post-irradiation. Nuclei were visualized with Hoechst 33342 staining. Representative photomicrographs (×1000 magnification) shown.

Studies have shown overlaps between phosphorylation of DNA-PKcs and γH2AX foci and phosphorylation of ATR and γH2AX foci [11, 21]. To verify the specific link between p-ATM and γH2AX, we observed a colocalization of γH2AX and p-ATM (Ser1987) foci at 1 h post-IR in MEF, DNA-PKcs^−/−^ MEF, and Ku70^−/−^ MEF cell lines (Figure 3A). We further investigated whether p-CHK1 (Ser345) and γH2AX colocalized. Our data showed that p-CHK1 (Ser345) was accumulated at DNA damage sites close to the site of γH2AX (Figure 3B). All of these data implied an initial role of ATM in DNA repair and the involvement of both the ATM–CHK2 and ATR–CHK1 pathways.

### Inactivation of core members of NHEJ in DSB repair implements cell cycle arrest

**Figure 4:**
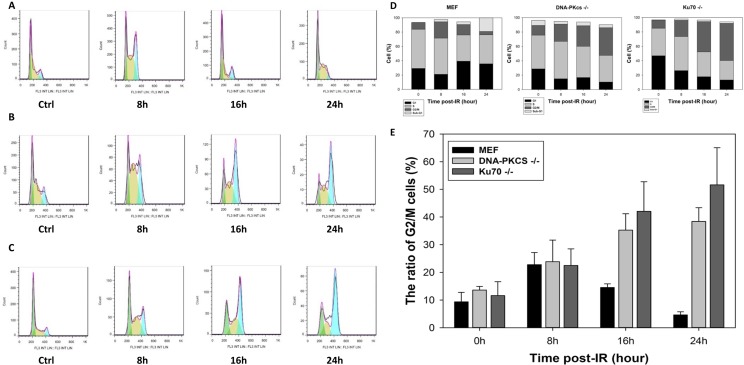
Deficiency in NHEJ repair results in cell cycle arrest Cell cycle distribution in asynchronous MEF (**A**), DNA-PKcs^−/−^ (**B**), and Ku70^−/−^ (**C**) cells at indicated times post-IR (5 Gy) was assayed by flow cytometric analysis (FACS) using propidium iodide (PI) staining for DNA content. The proportion of G1, S, and G2/M cells (**D**) and G2/M phase arrest (**E**) post-IR was determined. Data are means ± SD of the percentages of cells of three independent experiments.

To identify whether cell cycle arrest is biologically relevant to radiosensitivity, we performed a radiation-induced cell cycle profile of MEF, DNA-PKcs^−/−^ MEF, and Ku70^−/−^ MEF cell lines. Irradiation induced G2/M phase arrest in MEF cells at 8 h post-IR (Figure 4A, 4D), which was completely released at 16 h post-IR with a similar percentage of cells at the G2/M phase. However, irradiated DNA-PKcs^−/−^ and Ku70^−/−^ MEF cells exhibited a more robust and prolonged G2/M block in the cell cycle than did the wild-type MEF cells (Figure 4B–4D). The percentage of DNA-PKcs^−/−^ MEF cells in G2/M was 28.7% and 38.4% at 16 h and 24 h post-IR, respectively. A similar tendency was observed in Ku70^−/−^ MEF cells (Figure 4E). The G2/M arrest is the last major opportunity for preventing damaged DSBs from being taken into mitosis, which can result in mitotic catastrophe and cell death [22, 23]. Either DNA-PKcs or Ku70 knockdown cells could have a pronounced defect in DSB repair, thus leading to G2/M accumulation.

**Figure 5:**
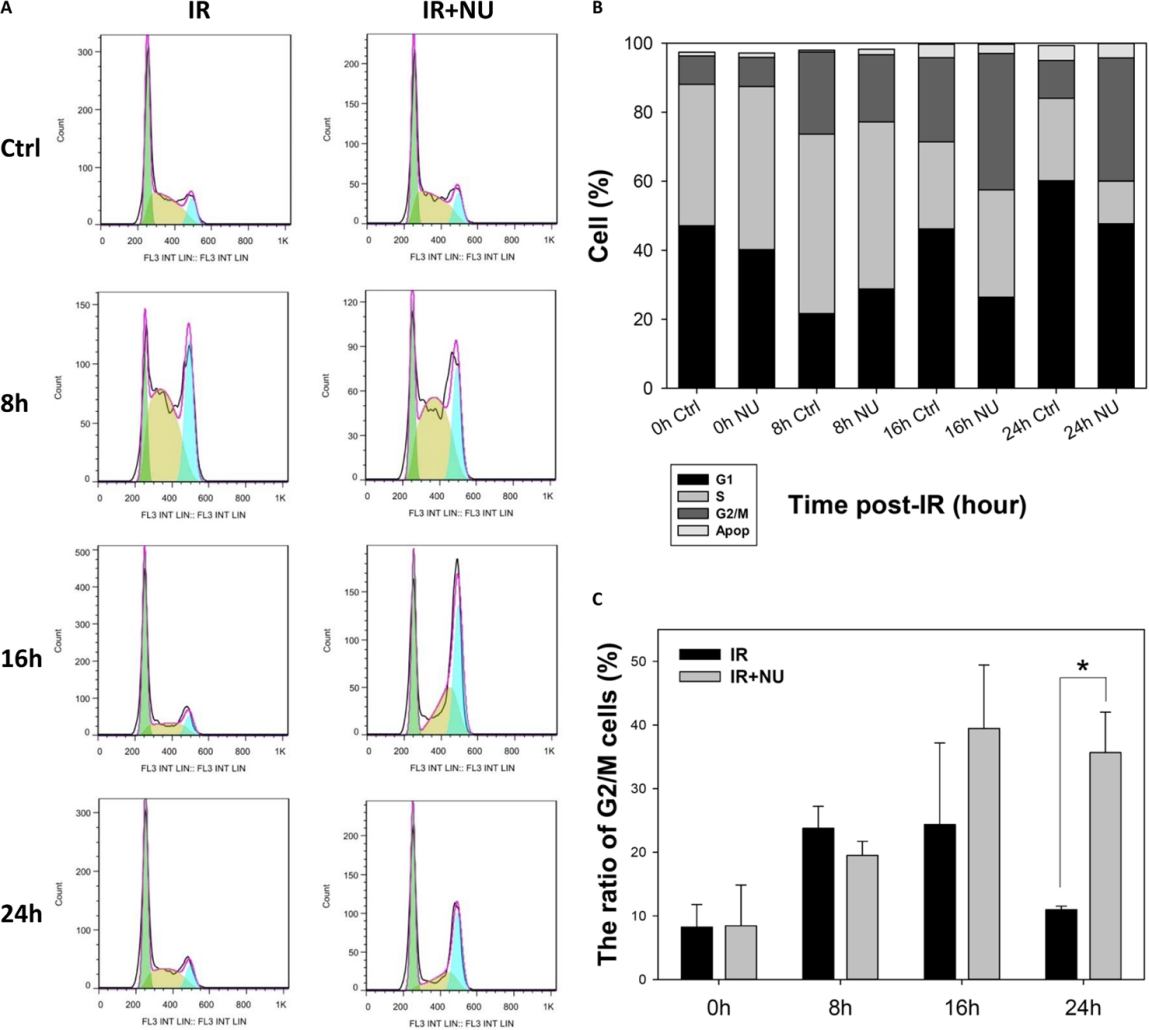
Inhibition of DNA-PKcs by NU7441 prolongs G2/M phase arrest (**A**) Cells were pretreated with 2 μM NU7441 for 1 h and exposed to 5 Gy IR. Cell cycle progression was monitored. (**B**) Cell cycle distribution in asynchronous MEF was measured at 8, 16, and 24 h post-IR. (**C**) Percentage of cells in G2/M phase. Data are means ± SD of the percentages of cells of three independent experiments.

To further examine the effect of DNA-PKcs inactivation on wild-type cells, we co-treated MEF cells with IR at 5 Gy and 2 μM NU7441. The population in G2/M phase was 23.8%, 24.4%, and 11.0% treated with IR alone at 8, 16, and 24 h post-IR; whereas a remarkable and permanent increase of 19.5%, 39.5%, and 35.7% in G2/M phase was detected with the combination of IR and NU7441 treatment at 8, 16, and 24 h post-IR, respectively (Figure 5A–5C); which were very similar to those for DNA-PKcs^−/−^ MEF cells. The results clearly and unequivocally confirmed the interaction between the cell cycle and DSB repair.

### The ATM–CHK2 pathway mediates IR-induced cell cycle arrest

**Figure 6:**
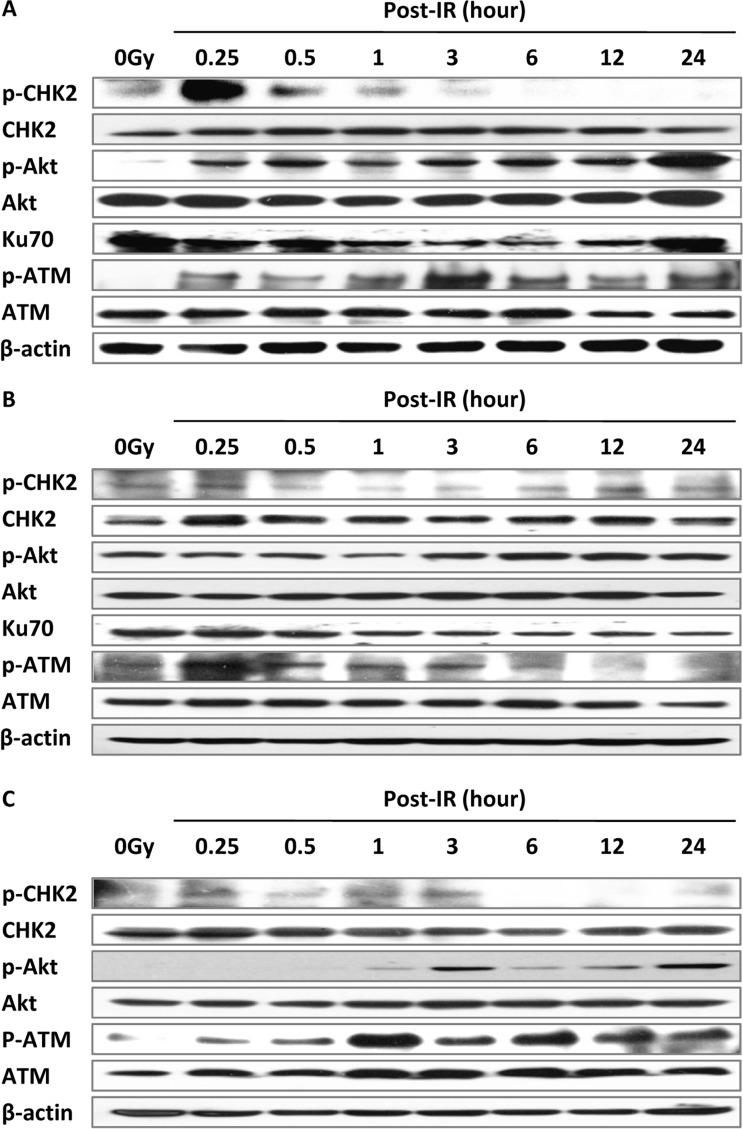
The ATM–CHK2 pathway interacts with the NHEJ repair pathway Irradiation at 5 Gy up-regulated cell cycle checkpoint signaling in MEF (**A**), DNA-PKcs^−/−^ (**B**), and Ku70^−/−^ (**C**) cells. Whole cell extract was prepared from untreated cells or collected at various times after irradiation and subjected to western blot analysis. β-actin was used as the loading control. Representative image from three independent experiments is shown.

The expression of ATM is activated by IR-induced DSBs and phosphorylates CHK2, which in turn triggers the G1 checkpoint [4]. CHK2 and CHK1 collaboratively function in G2/M phase arrest [8]. We performed a time course of activation of ATM–CHK2 pathway gene expression after treatment with irradiation in MEF, DNA-PKcs^−/−^ MEF and Ku70^−/−^ MEF cells. Phosphorylated ATM (serine 1987) appeared initially (at 15 min) and maintained a high level at 24 h post-IR in MEF and Ku70^−/−^ cells (Figure 6A, 6C) but returned to control level at 6 h post-IR in DNA-PKcs^−/−^ cells (Figure 6B). Our results showed a concordance with several studies reporting that inhibition of DNA-PKcs causes a reduction in IR-induced phosphorylation of ATM [16]. ATM initiated CHK2 activation, reached a maximum at 15 min post-IR, and then dropped to negligible levels at 3 h post-IR (Figure 6A, 6C) in MEF cells, which is consistent with the cell cycle trend of G2/M arrest being released after 8 h post-IR in MEF cells (Figure 4D). Of note, phosphorylation of CHK2 in DNA-PKcs^−/−^ MEF cells persisted to 24 h after treatment of irradiation (Figure 6B).

Akt is a downstream protein of PI3K in DDR and modulates G1/S and G2/M transitions [24, 25]. Gradually increasing expression of phospho-Akt (serine 473) after IR may be due to the cascade reaction. Taking these results together, we posited the dependency of IR-induced DSB repair on activation of cell cycle checkpoints.

### Activation of the ATR–CHK1 pathway plays a potential role in rejoining DNA DSB

**Figure 7:**
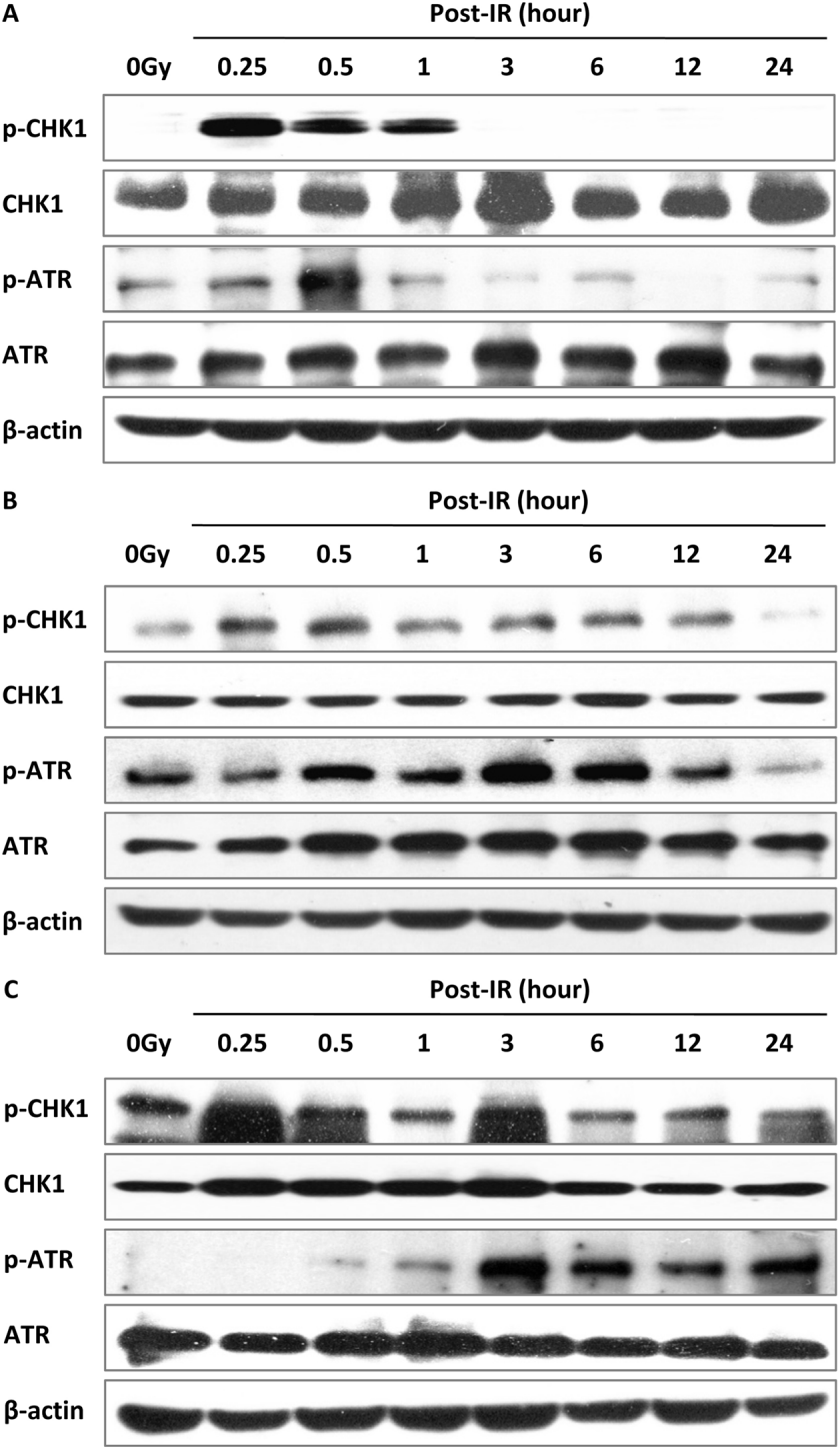
Activation of the ATR–CHK1 pathway is related to DSB repair ATR–CHK1 pathway proteins were activated post-IR (5 Gy) in MEF (**A**), DNA-PKcs^−/−^ (**B**), and Ku70^−/−^ (**C**) cells. Whole cell extract was prepared from untreated cells or collected at various times after irradiation and subjected to western blot analysis. β-actin was used as the loading control. Representative image from three independent experiments is shown.

ATR and its downstream effector CHK1 are hallmark mediators of intra-S phase and G2/M checkpoints, which collaborate to stabilize DSB and promote DNA repair [26–28]. In the present study, ATR was phosphorylated immediately after irradiation and declined in wild-type MEF cells while the level of p-ATR (S428) expression was sustained even at 24 h in DNA-PKcs^−/−^ and Ku70^−/−^ MEF cells (Figure 7A–7C). Similarly, phospho-CHK1 was triggered by IR and decreased at 3 h in MEF cells, and remained unchanged until 24 h after treatment in DNA-PKcs^−/−^ and Ku70^−/−^ MEF cells (Figure 7A–7C), indicating that it may still affect cell cycle arrest at 24 h after IR in cells deficient in the NHEJ pathway. The G2/M delay could result from the inability of DNA-PKcs^−/−^ or Ku70^−/−^ MEF cells to repair DSB, preventing their entry into mitosis in the cell cycle.

### Inhibition of DSB NHEJ repair mediates radiosensitivity in SUNE-1 cells

**Figure 8:**
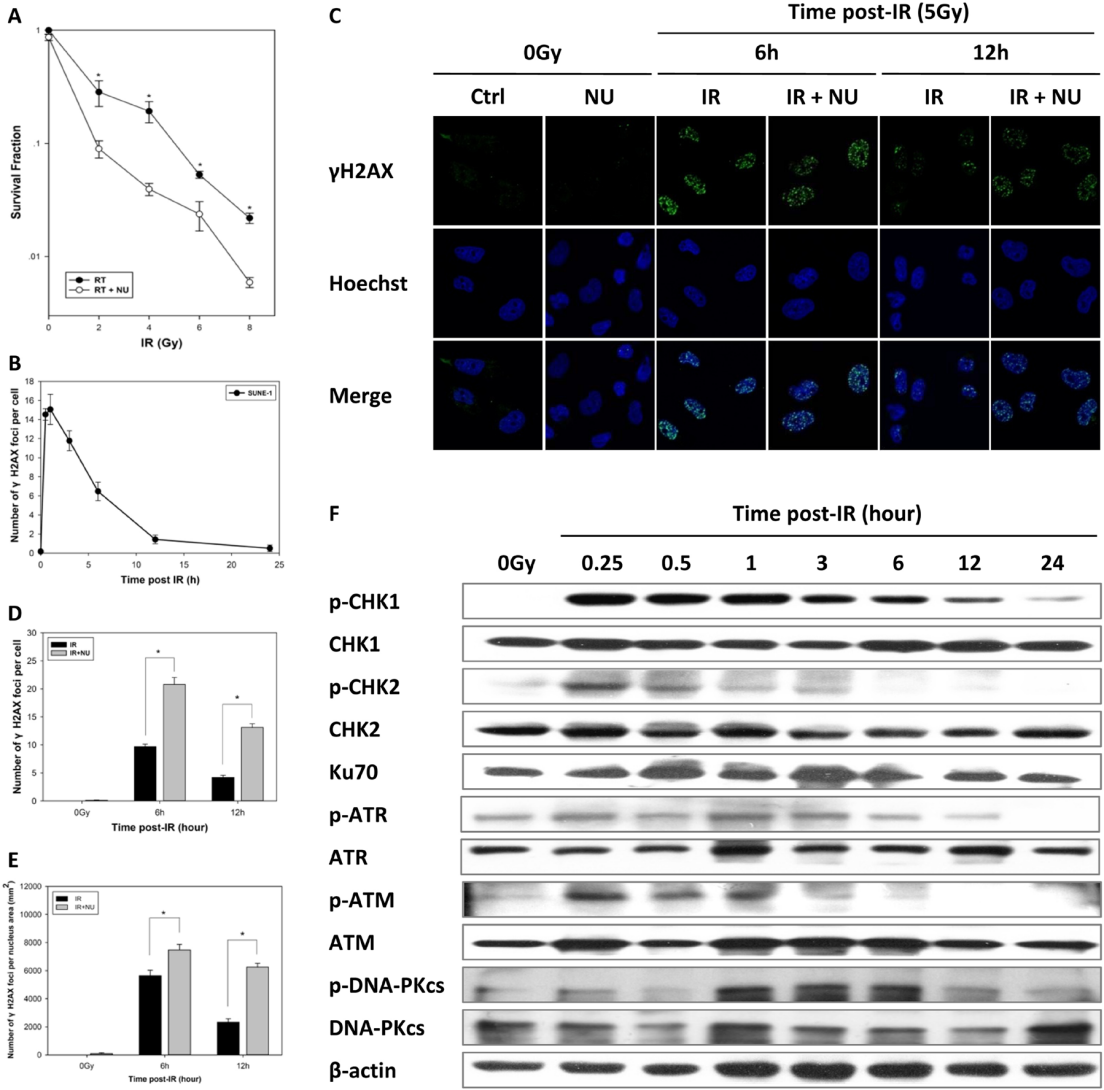
Inhibition of DNA repair induces radiosensitivity in nasopharyngeal carcinoma (**A**) Sensitization of nasopharyngeal carcinoma to IR was determined by clonogenic survival of SUNE-1 cells. Data are means ± SD from three independent experiments. (**B**) Average number of γH2AX foci per cell after exposure to 5 Gy IR in SUNE-1 cells, calculated using more than 30 cells. Data are mean ± SEM. (**C**) Attenuation of DNA-PKcs interfered with DNA repair. The cells were fixed at 6 or 12 h after treatment with IR at 5 Gy or a combination of IR and NU7441 (2 μM). Representative photomicrographs (×1000 magnification) are shown. Average number of foci per cell (**D**) and average number of foci per nucleus area (mm^2^) (**E**) were calculated by counting at least 30 nuclei. Data are mean ± SEM. (**F**) Expression of cell cycle checkpoint proteins analyzed by western blot. Whole cell extract was prepared from untreated cells or collected at specific times after irradiation.

Radiotherapy is currently the standard of treatment for NPC. Therefore, we studied SUNE-1 cells to test whether inactivation of DNA-PKcs sensitizes human NPC cells to IR. Our data showed that the combined treatment of irradiation with NU7441 significantly decreased cell viability (Figure 8A), confirming the role of DNA-PKcs in IR-induced cytotoxicity.

We further monitored the fate of DSBs after irradiation. DSBs were completely repaired until 24 h, and the kinetics in disappearance of γH2AX foci was observed to be similar by normalizing the quantified nuclear foci (Figure 8B). The combination of IR and NU7441 prolonged the persistence of γH2AX foci at relatively higher levels at 6 and 12 h post-IR when compared to irradiation alone (Figure 8C). We consistently quantified the number of foci per cell or per square millimeter, indicating significant γH2AX foci formation in the combined treatment group (Figure 8D–8E).

To investigate the role of cell cycle checkpoints in SUNE-1 cells after exposure to IR and the mechanism of DNA-PKcs-mediated radiosensitivity, we conducted a western blot analysis to examine the gene expression of the ATM–CHK2 and ATR–CHK1 signaling pathways. We found that the level of phospho-CHK2 reached a maximum at 15 min and fell to normal at 3 h post-IR which was accompanied by activation of ATM. Phosphorylation of ATR and phosphorylation of CHK1 persisted to 12 h post-IR (Figure 8F). The DNA-PKcs is phosphorylated by itself and ATM, which is crucial for NHEJ and cellular radioresistance [29]. Of interest, we discovered that phosphorylation of DNA-PKcs (S2056) was slower than that of ATM and phospho-DNA-PKcs persisted at a high level at 1 to 6 h post-IR (Figure 8F).

## DISCUSSION

We have found that a mechanism of DNA-PKcs activation mediated by cell cycle checkpoints is required for initiating the NHEJ repair pathway in response to irradiation. DNA-PKcs deficiency due to gene knockout or inhibition radiosensitizes MEF cells, impairs DSB repair, and prolongs cell cycle arrest. Inhibition of DNA-PKcs enhances sensitivity to irradiation in human NPC cells.

We previously confirmed that solid mouse tumors could be radiosensitized by heat-shock-induced expression of antisense Ku70 RNA, which inhibited expression of Ku70 [14]. Human glioma U-87 MG cells and colorectal carcinoma HCT-8 cells with depletion of the Ku70 NH_2_-terminal by a dominant-negative fragment of Ku70 (DNKu70) are vulnerable to irradiation [13]. All of these findings demonstrate that Ku70 is critical to tumor radiosensitivity *in vivo* and *in vitro*. In the present study, we have for the first time discovered the evidence that DNA-PKcs^−/−^ MEF cells exhibit radiosensitivity similar to Ku70^−/−^ MEF cells and that the addition of DNA-PK inhibitor leads to a modest effect on both, indicating that the role of DNA-PKcs in DSB repair after irradiation resembles that of Ku70 in NHEJ.

ATM, ATR, and DNA-PKcs are separately recruited to the radiation-induced DNA damage site by interaction with Nbs1, ATRIP, and Ku80 [30]. γH2AX is necessary to stimulate the DSB response to irradiation and is phosphorylated in the vicinity of DSBs by DNA-PKcs and ATM [29, 31, 32]. The manner of their phosphorylation overlaps [33, 34]. DNA-PKcs-deficient cells exhibit a higher level of γH2AX whereas the cells in the absence of ATM markedly suppress γH2AX formation [35, 36]. Our results have consistently revealed that irradiation induces more DSBs and that the velocity of DSB repair is significantly slower in DNA-PKcs^−/−^ MEF cells than in wild-type MEF cells. These results may suggest that a lack of DNA-PKcs has a modest effect on initiating DDR but severely interferes with DSB repair, which would influence genomic integrity.

Our previous data showed that DNA-PKcs selectively aborts apoptosis in mouse thymocytes whereas ATM disrupts G1/S cell cycle arrest [37]. Our present results indicate that the percentage of cells in G2/M phase was higher and that G2/M phase arrest persisted longer in Ku70^−/−^ MEF cells than in DNA-PKcs^−/−^ MEF cells, possibly in part due to activation of the G2/M cell cycle entry regulators ATR and CHK1. They are nearly dephosphorylated in DNA-PKcs^−/−^ MEF cells 24 h post-IR but are retained in Ku70^−/−^ MEF cells. The tumor suppressor protein p53, a downstream substrate of ATM, is accumulated during DDR and participates in cell cycle arrest [38, 39]. Phospho-p53 drops to normal levels at 8 h post-IR in DNA-PKcs^−/−^ MEF cells but persists to 16 h post-IR in Ku70^−/−^ MEF cells, indicating a role of Ku70/80 in activating the ATM and ATR signaling pathways [40]. This may explain our observation that the level of phospho-ATM is maximized at 1 h and minimized at 6 h post irradiation in DNA-PKcs^−/−^ MEF cells while the level of phospho-ATM expression were stabilized in Ku70^−/−^ MEF cells.

Our research has demonstrated that the level of γH2AX foci returns to the baseline at 6 h post-irradiation in wild-type MEF cells (Figure 2); whereas DSBs are present at a high level at 6 and 12 h and are completely repaired at 24 h post irradiation in tumor cells (Figure 8B). Blunt et al. identified a role of DNA-PKcs in V(D)J recombination and DSB repair, which functions as a radiosensitizer following irradiation [41]. NU7441 is a selective DNA-PK inhibitor which may sensitize cells to irradiation [42]. Preclinical studies have shown that NU7441 radiosensitizes hepatocellular carcinoma, prostate cancer, and breast cancer cell lines [43–45]; whereas its effect remains unclear in NPC cell lines. Of note, we have discovered the first evidence of a striking escalation of γH2AX foci in SUNE-1 cells treated with irradiation and NU7441 when compared with those treated with irradiation alone at 6 and 12 h. γH2AX foci almost disappeared in Ku70^−/−^ MEF cells 12 h after irradiation but persisted in DNA-PKcs^−/−^ MEF cells, indicating that suppression of DNA-PKcs activity can radiosensitize tumor cells rather than normal cells and might offer a promising therapy paradigm.

In conclusion, our results support the notion that radiosensitizing NHEJ repair-deficient cells is mediated by cell cycle checkpoints by delaying DNA repair, blocking cell cycle progression, and promoting apoptosis. NPC cells are susceptible to combined treatment with DNA-PK inhibitor and irradiation due to a resulting deficiency in DSB repair and activation of the cell cycle checkpoint. Cumulatively, the difference in DSB repair between normal and tumor cells in the suppression of DNA-PKcs activity offers promise for the development of new radiation-related therapeutic approaches.

## MATERIALS AND METHODS

### Cell lines and treatments

DNA-PKcs^−/−^, Ku70^−/−^, and wild-type mice on 129 genetic backgrounds were bred as previously reported [46, 47]. Mouse embryonic fibroblast (MEF) cells from DNA-PKcs^−/−^, Ku70^−/−^, and wild-type animals were isolated from 13.5-day-old mouse embryos, respectively. The genotype of the embryos was determined by PCR, which distinguished the endogenous from the targeted DNA-PKcs allele or Ku70 allele.

NU7441 (Tocris Bioscience), a DNA-PK inhibitor was dissolved in dimethylsulfoxide (DMSO) for 5 mmol/L stocks and stored at −20°C. All drugs were added to cells to a final DMSO concentration of 0.5%. Cells were exposed to X-rays generated by a RAD SOURCE RS2000 irradiator (Rad Source Technologies, Inc.) operating at 25 mA, with a 0.3-mm Al filter and effective photon energy of 160 kV. The dose rate at an irradiation distance of 48.6 cm was 1.31 Gy/min.

### Clonogenic survival assays

A total of 1.5 × 10^5^ of exponentially growing MEF, DNA-PKcs^−/−^ MEF, and Ku70^−/−^ MEF cells and SUNE1 cells were supplemented with control or NU7441 (1 μM)-containing medium for 1 h and then exposed to IR at 0, 2, 4, 6, or 8 Gy. After IR, the cells were incubated with or without NU7441 for a further 16 h. Cells were harvested and plated at 20–200 colonies per 100-mm dish in triplicate in drug-free medium and left to develop colonies. At 7–10 days later, colonies consisting of more than 50 cells were counted. After correction for plating efficiency, the data for survival colonies were used to plot clonogenic survival curves.

### Western blot analysis

Total protein and phosphorylated protein following IR were analyzed by Western blotting. Briefly, denatured protein (50 μg) was resolved by SDS-PAGE (6% or 10%) and then transferred onto a nitrocellulose membrane. Blots were blocked with 5% non-fat milk and incubated with specific primary antibodies overnight at 4°C. Primary antibodies against Akt (1:500), phospho-S473 Akt (1:500), CHK1 (1:500), phosphor-S345 CHK1 (1:500), CHK2 (1:500), ATR (1:500), phosphor-S428 ATR (1:500), ATM (1:500), and rabbit/mouse horseradish peroxidase-conjugated secondary antibodies (1:2000) were purchased from Cell Signaling Tech (CST). Anti-Ku70, anti-phospho-T68 CHK2, and anti-phospho-S2056 DNA-PKcs were from Abcam. Anti-phospho-S1987 ATM, anti-DNA-PKcs, and β-actin were from R&D, Santa Cruz, and Millipore, respectively. Bands were detected with ECL Western Blotting Substrate (Millipore).

### Immunofluorescence microscopy

For the foci assay, cells were seeded on coverslips (Electron Microscopy Sciences) for more than 24 h and exposed to IR at 5 Gy. To investigate the efficiency of NU7441, the cells were incubated with or without 2 μM NU7441 1 h before irradiation with 5 Gy X-ray and collected at the indicated time point. Cells were fixed with 4% paraformaldehyde, permeabilized with 0.2% Triton X-100, and blocked with 3% bovine serum albumin. The cover slips were incubated with the primary mouse monoclonal antibody for γH2AX (1:200, Millipore) overnight at 4°C. For visualizing colocalization, cells were collected 1 h post-irradiation and incubated with mouse anti-phospho-ATM antibody (Santa Cruz) and rabbit anti-γH2AX antibody (CST) or rabbit anti-phospho-CHK1 antibody (CST) and mouse anti-γH2AX antibody (Millipore). The cells were incubated with Alexa Fluor 488/555-conjugated secondary antibody (1:250, Life Technologies) for 1.5 h in the dark. Nuclei were stained with Hoechst 33342 (Sigma-Aldrich). Images were acquired by a LSM 710 laser-scanning confocal microscope (Zeiss), with foci counted in 30 cells and quantitative image analysis performed by ImageJ.

### Fluorescence-activated cell sorting (FACS)

For cell cycle analysis, cells were irradiated at 5 Gy in the presence or absence of 2 μM NU7441. Cells were then collected at specific time points by trypsinization and fixed with 70% ethanol at 4°C. Cellular DNA was labeled with propidium iodide (PI) staining solution (5 μg/mL PI, 250 μg/mL DNase-free RNase, 0.1% Triton X-100) for 30 min at 37°C. The distribution of cell cycle phases of at least 10,000 cells was determined using a FACS flow cytometer (Beckman, Gallios), and the proportion of cells at different phases was gated and calculated using Flowjo 7.6.1 software.

### Statistical analysis

The data were presented as the mean ± SD of at least three independent experiments. The results were tested for significance using the unpaired Student's *t* test by Sigma Plot 12.5 software.

## SUPPLEMENTARY FIGURE


