# Supplementary material for: Inhibiting DNA-PKcs in a non-homologous end-joining pathway in response to DNA double-strand breaks
Source: Oncotarget. 2017 Feb 7;8(14):22662–73. doi: 10.18632/oncotarget.15153 (PMC5410253; doi:10.18632/oncotarget.15153)
Supplement: Supplementary file 1 [file oncotarget-08-22662-s001.pdf]

Inhibiting DNA-PKcs in a non-homologous end-joining pathway in response to DNA double-strand breaks

Supplementary Materials

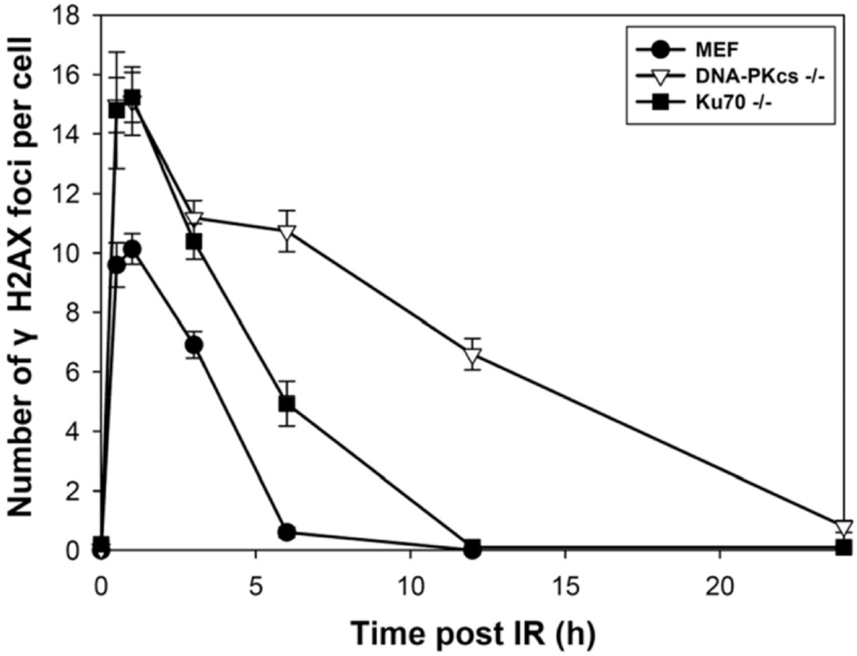

Supplementary Figure 1: Deficiency in the NHEJ pathway slows DNA repair. γH2AX foci in MEF, DNA-PKcs$^{-/-}$ MEF, and Ku70$^{-/-}$ MEF cells were quantified. Average number of foci per cell after exposure to 5 Gy IR was calculated using more than 30 cells. Data are mean ± SEM.
